# Prevalence and risk factors of *Helicobacter pylori* infection among children attending mercy pediatric hospital and royal hospital, Mogadishu, Somalia: a cross-sectional study

**DOI:** 10.3389/fped.2026.1918217

**Published:** 2026-08-17

**Authors:** Omar Sidow Zubair, Abdifitah Ibrahim Nor, Abdulwahed Nor Abdi, Abdikani Yasin Haji Ali, Farhia Mohamed Mire, Safia Mohamud, Nimco Mohamed Ahmed, Mohamed Hassan Mohamud, Dunia Haji Ali

**Affiliations:** 1 School of Postgraduate studies and research, Department of Tropical Infectious Disease Benadir University, Mogadishu, Somalia; 2 Department of Medical Laboratory Science, Faculty of Health Sciences, Mogadishu, Somalia; 3 Department of Public Health, Faculty of Health Sciences, University of Nairobi Faculty of Health Sciences, Nairobi, Kenya; 4 Faculty of Medicine and Surgery, Zamzam University of Science & Technology, Mogadishu, Somalia; 5 Faculty of Medicine and Health Science, University of Somalia, Mogadishu, Somalia

**Keywords:** children, cross-sectional study, helicobacter pylori, Mogadishu, prevalence, risk factors, Somalia, stool antigen test

## Abstract

**Background:**

*Helicobacter pylori* (*H*. *pylori*) infection is a major cause of peptic ulcer disease and gastric cancer, with the highest global prevalence in Africa. Despite this, pediatric data from Somalia remain critically scarce. This study determined the prevalence and independent risk factors of *H*. *pylori* infection among children attending two referral hospitals in Mogadishu, Somalia.

**Methods:**

A facility-based cross-sectional study was conducted from 14, February 2026 to 16, April, 2026 (two months) at Mercy Pediatric Hospital and Royal Hospital, Mogadishu. Sociodemographic and risk-factor data were collected through structured caregiver interviews, and *H*. *pylori* infection status was determined by stool antigen testing using an immunochromatographic assay. Univariable and multivariable logistic regression analyses were performed using SPSS version 20.0.

**Results:**

Using consecutive sampling, 175 children were enrolled (Mercy: 117; Royal: 58), 65 (37.1%; 95% CI: 30.0%–44.3%) were positive for *H*. *pylori* infection. On multivariable analysis, four factors were independently associated with infection: rural residence (aOR = 3.09; 95% CI: 1.20–7.96; *p* = 0.020), low parental education (aOR = 2.34; 95% CI: 1.03–5.35; *p* = 0.043), failure to wash fruits and vegetables before serving (aOR = 2.40; 95% CI: 1.06–5.46; *p* = 0.036), and street food consumption (aOR = 2.66; 95% CI: 1.29–5.51; *p* = 0.008). The model demonstrated good fit (Hosmer–Lemeshow *χ*^2^ = 5.44; *p* = 0.709). Soap use, which was strongly associated in univariable analysis, was attenuated to non-significance after multivariable adjustment, a pattern consistent with confounding.

**Conclusion:**

Approximately one in three children attending these referral hospitals was infected with *H*. *pylori*. Infection was independently associated with rural residence, lower parental education, and food-related exposures. Prevention strategies may benefit from system-level responses combining improved water and sanitation infrastructure, targeted caregiver education, and promotion of safe food-handling practices.

## Introduction

*Helicobacter pylori* (*H*. *pylori*) is a spiral-shaped, Gram-negative, microaerophilic bacterium that colonises the epithelial lining of the stomach and is accounts for the most common chronic bacterial infection in humans (1). Since its discovery as the causative agent of peptic ulcer disease in 1983, *H*. *pylori* have fundamentally altered the understanding of gastroduodenal pathology, transforming what was once a difficult, relapsing condition into one that is reliably curable with appropriate antibiotic therapy (1, 2). Beyond peptic ulceration, chronic *H*. pylori infection is causally associated with non-ulcer dyspepsia, gastric adenocarcinoma, and mucosa-associated lymphoid tissue (MALT) lymphoma—a spectrum of diseases that collectively impose a substantial global health burden (3, 4).

Globally, *H*. *pylori* infect approximately 48.5% of the world's population, with marked regional variation: 69.4% in South America, 54.6% in Asia, 47.0% in Europe, 37.1% in North America, and 79.1% in Africa (5). Africa carries the highest regional burden, with recent estimates placing continent-wide prevalence at 70.1% (6). This disproportionate burden has been attributed to the interplay of poverty, crowded living condition, inadequate access to clean water and sanitation, and limited healthcare infrastructure conditions that reported to favor faecal–oral and oral–oral transmission of the organism.

Infection with *H*. *pylori* is predominantly acquired in childhood, often before the age of five, and tends to persist lifelong in the absence of eradication therapy (7). Early acquisition is particularly important because it determines the duration of mucosal inflammation and thereby the risk of long-term sequelae. In developing nations, most children are infected in the first decade of life, with infection rates reflecting the socioeconomic and hygienic conditions of households and communities (8). A systematic review and meta-analysis of global pediatric studies found that *H*. *pylori* infection clustered in low-income settings and was significantly associated with crowding, poor water quality, and low parental education (9).

Across the African continent, prevalence data from children reveal consistently high infection rates, with notable variation: 65.7% among school children in Ethiopia, 21.8% in Kassala, eastern Sudan, and figures exceeding 80% in several Central African countries (10, 11). Within Somalia, the limited published evidence pertains predominantly to adult populations. Studies in Mogadishu have reported *H*. *pylori* prevalence of 47.2% among adults undergoing endoscopy, 56.2% among dyspeptic outpatients, and 42.7% among adults attending a medical outpatient clinic (12). Despite the recognized importance of early childhood infection as the foundation for adult disease burden, pediatric-specific data from Somalia are almost entirely absent from the literature.

This evidence gap is particularly important in the Somali context given the country's protracted health system fragility, widespread displacement, poor WASH infrastructure, and high rates of poverty—all factors consistently associated with H. pylori acquisition. Understanding the burden and determinants of pediatric *H*. *pylori* infection is therefore essential to inform targeted prevention, guide early detection programs, and shape caregiver education policies for this vulnerable age group. This study aimed to determine the prevalence of *H*. *pylori* infection and to identify the independently associated sociodemographic, environmental, and hygienic risk factors among children attending Mercy Pediatric Hospital and Royal Hospital in Mogadishu, Somalia.

## Methods

### Study design and setting

A facility-based, Cross-sectional study was conducted over a two-month period from 14, February 2026 to 16, April 2026 at two referral hospitals in Mogadishu, Somalia: Mercy Pediatric Hospital, located in the Warta Nabadda District, and Royal Hospital, located in the Hodan District. Both hospitals were selected for their wide catchment areas, high pediatric attendance volumes, and critical role in delivering primary and secondary pediatric care to diverse urban and peri-urban populations. A cross-sectional design was chosen as appropriate for estimating disease prevalence and examining associations between exposures and outcomes at a defined point in time—well suited to this descriptive-analytical objective in a resource-limited setting.

### Study population and eligibility criteria

The study population comprised children attending the outpatient pediatric departments of both hospitals during the data collection period. Children were eligible for inclusion if they were attending Mercy Pediatric Hospital or Royal Hospital and if their parents or legal guardians provided voluntary written informed consent.

Children were excluded if they had active watery diarrhea at the time of testing, had received antimicrobials, proton pump inhibitors, or bismuth-containing preparations within the four weeks preceding enrolment, or if their parents or guardians declined to consent.

### Sample size determination

The minimum required sample size was estimated using Cochran's formula for single-proportion prevalence studies: *n* *=* *Z*^2^* ×* *p*(1 − *p*)/*e*^2^. where *Z* = 1.96 (95% confidence level), *p* = 0.71 (71% prevalence, derived from a study conducted among gastritis patients in the Somali region of Ethiopia, the closest available geographic reference), and *e* = 0.05 (5% margin of error). This yielded an initial estimate of *n* = 316. Since the combined monthly pediatric attendance at both hospitals during the data collection period was estimated at approximately 390 children 2 months, a finite population correction was applied to account for the bounded sampling frame. With a combined two-month attendance estimate of *N* = 390, application of the finite population correction formula: *nf* *=* *n*/[1 *+* (*n* − 1)/*N*], yielded a corrected final sample size of *nf* = 175. Participants were allocated proportionally across the two hospitals based on their relative attendance volumes.

### Sampling procedure

A consecutive sampling approach was employed to enroll eligible participants throughout the two-month data collection period. Consecutive sampling, also known as systematic sequential enrolment, involves every eligible child who presents at the facility and meets all inclusion criteria, without randomization or selection, until the required sample size is reached for each site. This method was selected because it is non-selective, eliminates volunteer and convenience bias, and is highly feasible in a busy outpatient setting where exhaustive enrolment of all eligible attendees can be maintained by trained research staff.

At each hospital, a research staff member was present during all outpatient clinic sessions throughout the study period. Every child presenting for care was screened against the inclusion and exclusion criteria. All eligible children were approached sequentially, and their caregivers were invited to participate. Following informed consent, the questionnaire was administered and a stool sample was collected from each enrolled participant. Enrolment continued daily until the site-specific sample size target was reached.

### Data collection instruments

#### Structured questionnaire

Sociodemographic characteristics and potential risk factor data were collected through face-to-face structured interviews with parents or legal guardians, conducted by trained data collectors. The questionnaire captured child age, sex, and place of residence; parental educational attainment and housing tenure status; household water source, toilet facility type, number of rooms, and household size; and hygiene and dietary practices including water treatment, handwashing frequency and technique, soap use, produce washing, raw food consumption, and street food eating patterns. The questionnaire was pre-tested on approximately 5%–10% of the anticipated sample size at a comparable facility not included in the study, and revisions were made to improve clarity and contextual appropriateness before main data collection commenced. Interviews were conducted in Somali with the assistance of bilingual research staff.

#### Laboratory test

*H. pylori* infection was diagnosed by stool antigen testing using a one-step immunochromatographic (lateral-flow, colloidal-gold) *H. pylori* stool antigen test (SAT) (H. pylori Antigen Rapid Test Device [Feces], EvanCare; Precmon Medical [Nantong] Co., Ltd, Nantong, Jiangsu, China). This non-invasive assay is a recommended primary diagnostic method for *H. pylori* in young children, as it detects current, active infection and avoids the need for invasive endoscopic procedures; the manufacturer reports a sensitivity of 97.0%, specificity of 98.5%, and accuracy of 99.0% relative to endoscopy-based methods. Fresh stool samples were collected from each child in sterile, labelled, leak-proof containers, and caregivers received standardized verbal and written instructions on proper sample collection and timely submission. All sample handling and testing were performed strictly according to the manufacturer's instructions: test kits were stored at 4–30 °C, and specimens not tested immediately were refrigerated at 2–8 °C and processed within 7 days. For each test, three drops (∼150 µL) of the extracted sample were dispensed into the specimen well, and results were read at 10 min (and not later than 15 min) by two independent readers blinded to participants' clinical data; discordant results were resolved by a third reader. A result was interpreted as positive when both a test line (T) and a control line (C) were present, negative when only the control line (C) appeared, and invalid when the control line was absent, in which case testing was repeated on a new device.

### Quality control

#### Questionnaire

Content and face validity were established through review by the researcher prior to data collection. All data collectors received a minimum of one day of standardized training using a research script and mock interview exercises before commencing enrolment. Completed questionnaires were reviewed on the same day of administration by a supervisor for completeness, logical consistency, and adherence to skip patterns. A random 5% subsample of questionnaires was re-administered within 24–48 h to assess inter-rater agreement. Double data entry with automated discrepancy checks was applied during data entry into SPSS.

#### Laboratory

Pre-analytical quality control measures included collection of samples in sterile leak-proof containers with rejection criteria for urine contamination or insufficient volume. Temperature logs were maintained for specimen transport and storage. On each testing day, external positive and negative control specimens were run and documented before patient samples were processed. Kit lot numbers and expiry dates were recorded in a dedicated quality control log. All cassettes were read within the manufacturer's specified time window and by two blinded readers, with a third reader adjudicating discordance. A random 5%–10% subset of specimens was retested in duplicate to monitor reproducibility. Standard biosafety precautions—including personal protective equipment, surface decontamination, and biohazard waste disposal—were enforced throughout.

### Data management and statistical analysis

All questionnaire data were entered into using IBM SPSS Statistics version 26 (IBM Corp., Armonk, NY, USA*.* All 175 enrolled children had complete data for the outcome variable and for every sociodemographic, environmental, hygienic, and dietary covariate; there were no missing observations, and all analyses were therefore performed on the full sample (*N* = 175) without imputation or case exclusion. Descriptive statistics were computed as frequencies and percentages for all categorical variables. The overall prevalence of H. pylori infection was estimated as a proportion with a 95% Wald confidence interval. Associations between potential risk factors and *H*. *pylori* antigen test result were first examined using univariable logistic regression, with results expressed as crude odds ratios (cOR) and 95% confidence intervals. Variables with an overall model *p*-value of less than 0.20 at the univariable level were considered candidates for inclusion in the multivariable model—a threshold widely adopted in epidemiological modelling to ensure that variables with clinically plausible associations are not prematurely excluded by the conservative *α* = 0.05 criterion.

Multivariable binary logistic regression was then performed. Variable selection for the final multivariable model was guided by the 10-events-per-variable (EPV) rule: with 65 positive cases, the model could accommodate a maximum of six to seven independent parameters. Collinear or conceptually overlapping variables were excluded after cross-tabulation inspection and theoretical review. The final model included residence type, parental education, produce washing, street food consumption, soap use, household size, and age category. Model fit was assessed using the Hosmer–Lemeshow goodness-of-fit test, with *p* > 0.05 indicating adequate fit. Results are presented as adjusted odds ratios (aOR) with 95% confidence intervals. Statistical significance was defined at *p* < 0.05 throughout.

### Ethical considerations

Ethical approval was obtained from the National Institute of Health, Somalia (NIH) under approval number NIH/SNREB006/FEB/2026. All procedures were conducted in accordance with the Declaration of Helsinki. Voluntary written informed consent was obtained from the parent or legal guardian of each child prior to enrolment. Participants were informed of the study purpose, procedures, voluntary nature of participation, and right to withdraw without consequence. Participant confidentiality was protected by assigning unique study identification codes to all data. No personally identifiable information was linked to stool samples or questionnaire data beyond the research team. Children identified as positive for *H*. *pylori* infection were referred to the attending clinician for appropriate clinical management.

## Results

### Sociodemographic characteristics of participants

A total of 175 children were enrolled across the two study sites during the two-month data collection period, achieving a 100% response rate consistent with the consecutive sampling approach. The sociodemographic, hygienic, and dietary characteristics of the study cohort are summarized in Table 1. Of the enrolled children, 101 (57.7%) were male and 74 (42.3%) were female. The majority (100; 57.1%) were aged under five years, with a further 66 (37.7%) aged five to ten years and nine (5.1%) aged ten to eighteen years. Most children resided in urban areas (144; 82.3%). With respect to parental education, 122 caregivers (69.7%) had attained only primary-level schooling or less, while 53 (30.3%) had completed secondary education or above. The predominant housing tenure arrangement was rental accommodation (106; 60.6%).

**Table 1:** Sociodemographic, and hygienic characteristics of the study cohort (*N* = 175).

Variable	Category	Frequency (*n*)	Percentage %
Child's sex	Female	74	42.3
	Male	101	57.7
Age category	Under 5 years	100	57.1
	5–10 years	66	37.7
	10–18 years	9	5.1
Type of residence	Rural	31	17.7
	Urban	144	82.3
Parental education	Primary or less	122	69.7
	Above secondary	53	30.3
Housing tenure status	Rent for money	106	60.6
	Owned	53	30.3
	Occupied without rent	16	9.1
Primary household water source	Tap water	110	62.9
	Bottled water	50	28.6
	Borehole water	15	8.6
Type of toilet facility	Pit latrine/Others	91	52.0
	Flush toilet	74	42.3
	Open defecation	10	5.7
Number of rooms in house	4–6 rooms	87	49.7
	1–3 rooms	69	39.4
	No rooms (IDPs)	19	10.9
Household size	4–6 members	98	56.0
	≥7 members	49	28.0
	1–3 members	28	16.0
Practices water treatment	No	130	74.3
	Yes	45	25.7
Hand washing after toilet	Yes	167	95.4
	No	8	4.6
Hand washing before meals	Yes	140	80.0
	No	35	20.0
Uses soap for hand washing	Yes	94	53.7
	No	81	46.3
Washes fruits/vegetables	Yes	119	68.0
	No	56	32.0
Consumes raw/undercooked food	No	161	92.0
	Yes	14	8.0
Eats street food	No	102	58.3
	Yes	73	41.7
Total	175	100.0	

IDPs, internally displaced persons.

### Hygiene and dietary practices

In terms of hygiene and dietary practices, 130 households (74.3%) reported not treating drinking water before consumption. Hand washing after toilet use was reported by 167 caregivers (95.4%) and before meals by 140 (80.0%), though soap use was reported by only 94 respondents (53.7%). Fifty-six caregivers (32.0%) reported not washing fruits and vegetables before serving, and 73 children (41.7%) were reported to consume street food.

### Prevalence of H. pylori infection

As shown in Table 2, 65 of the 175 children tested (37.1%; 95% CI: 30.0%–44.3%) were positive for *H. pylori* by stool antigen test. This indicates that approximately one in three children attending these referral hospitals harbored active *H. pylori* infection at the time of testing.

**Table 2:** Univariable logistic regression analysis of factors associated with *H*. *pylori* infection (*N* = 175).

Variable	Category	cOR	95% CI	*p*-value
Child's sex	Male (Ref)	1.00	—	0.632
Female	0.63–2.16			
Age category	Under 5 years (Ref)	1.00	—	0.210
5–10 years	0.93–3.36			
10–18 years	0.25–4.52			
Type of residence	Urban (Ref)	1.00	—	0.001**
Rural	1.77–9.04			
Parental education	Above secondary (Ref)	1.00	—	0.002**
Primary or less	1.43–6.44			
Housing tenure status	Rent for money (Ref)	1.00	—	0.047*
Occupied without rent	1.02–8.68			
Owned	0.97–3.78			
Primary water source	Bottled water (Ref)	1.00	—	0.039*
Borehole water	1.36–16.00			
Tap water	0.65–2.74			
Type of toilet facility	Flush toilet (Ref)	1.00	—	0.055
Open defecation	0.46–7.05			
Pit latrine/others	1.15–4.28			
Number of rooms	1–3 rooms (Ref)	1.00	—	0.123
4–6 rooms	0.64–2.42			
No rooms (IDPs)	1.04–8.33			
Household size	1–3 people (Ref)	1.00	—	0.064
4–6 people	0.40–2.40			
≥7 people	0.83–5.81			
Water treatment	Yes (Ref)	1.00	—	0.537
No	0.61–2.55			
Handwashing after toilet	Yes (Ref)	1.00	—	0.449
No	0.42–7.20			
Handwashing before meals	Yes (Ref)	1.00	—	0.054
No	0.99–4.44			
Use of soap	Yes (Ref)	1.00	—	0.005**
No	1.30–4.55			
Washes fruits/vegetables	Yes (Ref)	1.00	—	0.001**
No	1.60–6.00			
Consumes raw food	Yes (Ref)	1.00	—	0.908
No	0.34–3.34			
Eats street food	No (Ref)	1.00	—	0.013*
Yes	1.18–4.14			

cOR, crude odds ratio; CI, confidence interval; Ref, reference category. *p < 0.05; **p < 0.01. Variables with overall model *p* < 0.20 were advanced to multivariable analysis.

### Factors associated with *H. pylori* infection on univariable analysis

Univariable logistic regression was performed for all fifteen candidate explanatory variables, with results presented in Table 3. Using an overall model *p*-value threshold of less than 0.20 as the criterion for advancement to multivariable analysis, eleven variables qualified: type of residence (*p* = 0.001), parental education (*p* = 0.002), housing tenure status (*p* = 0.047), primary water source (*p* = 0.039), type of toilet facility (*p* = 0.055), number of rooms (*p* = 0.123), household size (*p* = 0.064), handwashing before meals (*p* = 0.054), soap use (*p* = 0.005), produce washing (*p* = 0.001), and street food consumption (*p* = 0.013). Child sex, age category, water treatment practices, handwashing after toilet use, and consumption of raw food did not meet the threshold and were not advanced. Of the qualifying variables, rural residence (cOR = 4.00; 95% CI: 1.77–9.04), borehole water use (cOR = 4.67; 95% CI: 1.36–16.00), low parental education (cOR = 3.03; 95% CI: 1.43–6.44), failure to wash produce (cOR = 3.10; 95% CI: 1.60–6.00), and absence of soap use (cOR = 2.43; 95% CI: 1.30–4.55) were the strongest crude predictors of infection.

**Table 3:** Multivariable logistic regression analysis of independent factors associated with *H*. *pylori* infection (*N* = 175).

Variable	Category	aOR	95% CI	*p*-value
Type of residence	Urban (Ref)	1.00	—	0.020*
Rural	3.09	1.20–7.96		
Parental education	Above secondary (Ref)	1.00	—	0.043*
Primary or less	2.34	1.03–5.35		
Washes fruits/vegetables	Yes (Ref)	1.00	—	0.036*
No	2.40	1.06–5.46		
Eats street food	No (Ref)	1.00	—	0.008**
Yes	2.66	1.29–5.51		
Use of soap	Yes (Ref)	1.00	—	0.710
No	1.16	0.53–2.57		
Household size	1–3 people (Ref)	1.00	—	0.208
4–6 people	1.18	0.43–3.30		
≥7 people	2.27	0.74–6.96		
Age category	10–18 years (Ref)	1.00	—	0.339
Under 5 years	1.66	0.34–8.07		
5–10 years	2.57	0.52–12.65		

aOR, adjusted odds ratio; CI, confidence interval; Ref, reference category. *p < 0.05; **p < 0.01. Model: likelihood ratio *χ*^2^ = 38.36, df = 9, *p* < 0.001; Nagelkerke *R*^2^ = 0.166; Hosmer–Lemeshow *χ*^2^ = 5.44, df = 8, *p* = 0.709.

### Independent predictors of H. pylori infection on multivariable analysis

The final multivariable model, detailed in Table 3, included seven predictors across nine parameters, remaining within the 10-events-per-variable (EPV) limit for 65 positive cases. The overall model was highly significant (likelihood ratio *χ*^2^ = 38.36; df = 9; *p* < 0.001), with a Nagelkerke pseudo-*R*^2^ of 0.166. The Hosmer–Lemeshow goodness-of-fit test yielded *χ*^2^ = 5.44, df = 8, *p* = 0.709, indicating adequate model fit.

Four variables retained independent statistical significance after adjustment. Rural residence showed the strongest independent association, with rural children having approximately three times the odds of infection compared to urban children (aOR = 3.09; 95% CI: 1.20–7.96; *p* = 0.020). Children of parents with primary-level education or less had more than twice the adjusted odds of infection compared to children of more highly educated parents (aOR = 2.34; 95% CI: 1.03–5.35; *p* = 0.043). Failure to wash fruits and vegetables before serving was independently associated with more than twice the odds of infection (aOR = 2.40; 95% CI: 1.06–5.46; *p* = 0.036).

Street food consumption showed the most robust behavioural association, with the estimate strengthening after adjustment (cOR = 2.21 to aOR = 2.66; 95% CI: 1.29–5.51; *p* = 0.008) indicating that the unmonitored commercial food environment constitutes an independent exposure pathway uncoupled from domestic hygiene.

Notably, soap use for handwashing—which appeared strongly protective in univariable analysis (cOR = 2.43; *p* = 0.005)—lost statistical significance entirely after multivariable adjustment (aOR = 1.16; 95% CI: 0.53–2.57; *p* = 0.710). This substantial attenuation demonstrates that the crude association was heavily confounded by structural factors, particularly rural residence, low parental education, and food-related exposures. In this population, soap non-use appears to function as a downstream indicator of socioeconomic and geographic disadvantage rather than showing an independent association with infection. Household size and age category did not achieve independent significance but were retained in the model for confounding control and to maintain mathematical stability.

## Discussion

This cross-sectional study found a prevalence of *H. pylori* infection of 37.1% (95% CI: 30.0%–44.3%) among 175 children attending two referral hospitals in Mogadishu, Somalia, with rural residence, low parental education, failure to wash fruits and vegetables, and street food consumption emerging as independent determinants. These findings provide the first child-specific, analytically robust *H. pylori* prevalence data from Mogadishu, contributing to a sparse evidence base from this fragile health system setting.

The observed prevalence aligns closely with the pooled stool-antigen prevalence of 34.64% (95% CI: 28.71–40.58%) reported in a recent East African systematic review and meta-analysis (13), reinforcing the validity of the present estimate. It is notably lower than the adult prevalence reported in Somalia itself (42.7%–56.2%) (14). Because *H. pylori* is predominantly acquired in early childhood and infection typically persists thereafter, this age-related difference is best interpreted as a birth-cohort effect rather than continued acquisition with age: today's adults were children under poorer water, sanitation, and housing conditions and therefore experienced higher childhood transmission, whereas the lower prevalence among the present cohort likely reflects gradual improvements in these conditions over time (7–15). The estimate is also lower than rates recorded among Ethiopian school-age children and gastritis patients (65.7%–71.0%) (16, 17).

Rural residence was the strongest independent predictor of infection (aOR = 3.09; *p* = 0.020). Although the crude estimate was larger (cOR = 4.00), the persistence of a substantial adjusted effect indicates that the elevated odds among rural children appear to be associated with structural and infrastructural factors rather than with individual behavioral consistent with the well-documented urban–rural gradient in *H. pylori* epidemiology, attributable to limited potable water access, inadequate sanitation, and crowded living conditions (18). In the present data, borehole water reliance was almost entirely confined to rural households, supporting the interpretation of residence as an upstream proxy for a cluster of environmental disadvantages. Comparable rural predominance has been reported among Nigerian children, where rural residence was likewise associated with poor water supply, deficient sanitation, low education, and poverty (17).

Low parental educational attainment was a second independent determinant (aOR = 2.34; *p* = 0.043), functioning as a proxy for household health literacy and shaping caregivers' awareness of faecal–oral and oral–oral transmission routes. Its persistence after adjustment for residence and hygiene behaviours indicates that the influence of parental schooling is not entirely mediated by material conditions, but partly reflects independent differences in knowledge, preventive behaviour, and care-seeking patterns (18). This is concordant with international evidence identifying low parental education as a recognised pediatric *H. pylori* risk factor across diverse settings (8–19).

The two dietary variables retained in the final model highlight food as an active transmission vehicle in this community. Raw produce irrigated with untreated or sewage-polluted water is a well-established vehicle for *H. pylori* transmission, and unwashed vegetables have been specifically catalogued among pediatric risk factors in the global literature (8–16). Street food vendors typically operate without continuous running water or dedicated sinks, conditions documented as probable mechanisms of transmission in developing settings (18, 19). Notably, the street food association strengthened under adjustment (cOR = 2.21 to aOR = 2.66; *p* = 0.008) a methodologically important finding, as it confirms that this effect is not simply a proxy for domestic poverty or poor hygiene at home but represents a genuinely independent, additive exposure.

By contrast, the attenuation of soap use to non-significance under adjustment (cOR = 2.43 to aOR = 1.16; *p* = 0.710) carries direct implications for prevention messaging. In this population, soap non-use appears to act not as a standalone biological driver but as a marker for families living in higher-exposure, lower-resource environments, where rural infrastructure, food contamination, and low health literacy operate independently and more powerfully. This contrast underscores the necessity of multivariable modelling to isolate true independent drivers, and cautions against promoting soap use in isolation while structural and food-borne determinants remain unaddressed (20).

Several limitations should be considered when interpreting these findings. First, participants were recruited consecutively from the outpatient departments of two hospitals, and hospital-based sampling may introduce selection bias, as children presenting to referral facilities are likely to differ systematically from the general child population with respect to symptom severity, comorbidity burden, and caregiver health-seeking behavior. The prevalence observed in this study should therefore be regarded as a facility-based estimate rather than a community estimate. Second, the cross-sectional design captured *H. pylori* status and exposures simultaneously and therefore permits assessment of associations but not causal or temporal inference; the identified determinants cannot be confirmed to have preceded infection. finally, although the multivariable model adjusted for residence, parental education, household size, age, and key hygiene and dietary practices, residual confounding cannot be excluded, as unmeasured or imprecisely measured factors—including household income, intrafamilial *H. pylori* status, breastfeeding history, prior antibiotic exposure, and detailed WASH characteristics—may have influenced the observed associations. Related to this, exposure and risk-factor data were obtained by caregiver self-report and are subject to recall and social-desirability bias, which may have led to non-differential misclassification and attenuation of some effect estimates. Finally, the modest Nagelkerke pseudo-*R*^2^ (0.166) indicates that a substantial proportion of the variance in infection status remains unexplained by the measured covariates. Despite these limitations, the study provides the first child-specific prevalence and risk-factor data for *H. pylori* infection in Mogadishu and offers a useful baseline for future community-based, longitudinal, and multi-site investigations.

This study has several limitations that should be considered when interpreting the findings. First, the cross-sectional design captures H. pylori status at a single point in time and therefore cannot establish causal or temporal relationships between the identified factors and infection. Second, the study was conducted in two facilities in Mogadishu, so the findings may not be generalisable to children in community or rural settings. Third, exposure and risk-factor data were based on caregiver self-report and may be subject to recall and social-desirability bias. Despite these limitations, the study provides useful baseline data on the prevalence of H. pylori infection and its associated factors among children in this setting.

## Conclusion

This study found a prevalence of 37.1% for *H*. *pylori* infection among children attending referral hospitals in Mogadishu, Somalia equivalent to more than one in three children already harboring active infection. After multivariable adjustment, four independent determinants were identified: rural residence, low parental education, failure to wash fruits and vegetables before serving, and street food consumption. The collapse of the soap-use association under adjustment suggests that H. pylori infection in this setting is more strongly associated with structural, socioeconomic, and food-related factors than with isolated personal hygiene behaviours.

Reducing the childhood H. pylori burden may require interventions that extend beyond individual hygiene promotion to address the structural and food-related factors identified in this study.

Priority actions include improving water supply and sanitation infrastructure in rural and peri-urban areas; delivering targeted, literacy-appropriate caregiver health education—particularly in households with low parental education; and promoting safe food-handling practices including thorough washing of fruits and vegetables and awareness of risks associated with unregulated street food. Early screening and management of symptomatic children attending pediatric services should be integrated into routine clinical practice. Future research should employ community-based, longitudinal, multi-site designs with confirmatory diagnostic methods to establish temporal relationships, quantify intrafamilial transmission dynamics, and evaluate the effectiveness of targeted interventions in this population.

## Data Availability

The original contributions presented in the study are included in the article/Supplementary Material, further inquiries can be directed to the corresponding author/s.
